# Canine detection for biodiversity protection: a multispecies approach to promote conservation and prevent wildlife trafficking

**DOI:** 10.3389/fvets.2026.1816996

**Published:** 2026-05-13

**Authors:** Jorge U. Rojas-Guevara, Paola A. Prada-Tiedemann, Jhon Buenhombre, María N. Cajiao

**Affiliations:** 1 Animal Sciences Research Group, Animal Welfare and Ethology Concentration, College of Agricultural Sciences, Fundación Universitaria Agraria de Colombia – UNIAGRARIA, Bogotá, Colombia; 2 Forensic Analytical Chemistry and Odor Profiling Laboratory, Department of Environmental Toxicology, Texas Tech University, Lubbock, TX, United States

**Keywords:** animal trafficking, chemical signals, detector dogs, odor discrimination, olfactory memory, standards working dogs, wildlife trade

## Abstract

The Colombian National Police (CNP) has canine teams specialized for detecting mammals, reptiles, and birds illegally trafficked by national and transnational criminal organizations. The objectives are to: (1) establish standards for the scent database, training protocol, and certification of canine teams for wildlife detection; and (2) determine dog sensitivity and specificity in discriminating between target and decoy scents through a double-blind test during the certification of animal scent detection dogs. The methodology initially involved 22 dogs; a structured screening process, based on behavioral suitability, trainability, and preliminary scent discrimination performance, reduced the cohort to four dogs that met the required operational criteria. These dogs conducted three tests daily for 3 days, to discriminate between target and decoy scents, including horsehair, dog and cat food, pig hooves, beef, and chicken feathers. A double-blind test was performed to assess evaluator agreement (Cohen’s Kappa), as well as diagnostic performance, where sensitivity was defined as the proportion of correctly identified true positives (excluding false-negatives) and specificity as the proportion of correctly rejected true negatives (excluding false-positives), with false-positives and false-negatives recorded accordingly. The four dogs achieved near-perfect agreement (0.95–1.00) for the scents of feathers from the *Ognorhynchus icterotis* and *Ara macao*; hairs from the *Saimiri sciureus*; carapace of the *Trachemys callirostris*; skin from the *Boa constrictor*; and scales from *Crocodylus fuscus* and *Iguana iguana*. False positives for the pig-hoof scent as a decoy occurred on the first evaluation day for two of the dogs, but were not present on subsequent days. The established protocol was successful in training and certifying dogs for wildlife odor detection, with sensitivity and specificity ranging from 0.95 to 1.00 across all tested odors. Additional scents should be incorporated during training to achieve high success rates during certification and subsequent performance. Training materials should be species-specific to avoid contamination with other scents. The established protocol was effective for training and certifying canine teams for wildlife odor detection, demonstrating high levels of agreement, sensitivity, and specificity during controlled testing conditions. These findings support the use of standardized, species-specific scent materials and double-blind evaluation methods in certification processes for wildlife-detection dogs.

## Graphical abstract

**Figure:**
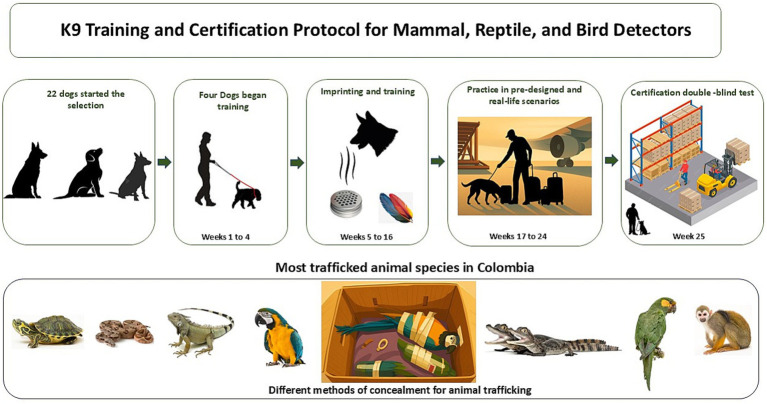

## 1 Introduction

Conservation detection dogs (CDDs) have emerged as one of the most effective and versatile tools in wildlife conservation and anti-trafficking efforts. Their exceptional olfactory capabilities enable them to locate elusive species, biological traces (such as scat, feathers, or other scent material), and even wildlife products destined for illegal trade, often outperforming traditional survey methods (1). For example, in a field study targeting an endangered rabbit species, a scent-detection dog trained on limited scent samples achieved 98% specificity and successfully detected several live individuals in the wild (2). Moreover, in international primate conservation research, detection dogs located non-human primate fecal samples with 92% accuracy—significantly outperforming human-only teams under challenging field conditions (3).

CDDs’ efficacy rates have been studied across a wide range of endangered species (4), including applications in bear wildlife monitoring (5), bat carcass detection at wind turbine sites (6), and surveys of aquatic mammals (7), as well as birds (8), reptiles (9), amphibians (10), and insects (11). These methods have been used in 62 countries across more than 480 species (12). Additionally, CDDs can detect more samples, are faster, and cover greater distances than other methods, especially for locating excrement and indicating the presence of animals in conjunction with other analytical techniques such as DNA (13, 14).

In Colombia, canine units have long been employed within a wide range of law enforcement control systems across various specialties, including narcotics, explosives, antipersonnel mine detectors, improvised explosive devices (IEDs), unexploded ordnance, oil leaks, tracking people, and currency detection (15). Following the rise in illicit crop cultivation after peace agreements with drug-trafficking groups (16), the Colombian National Police (CNP) has adapted its detector–dog standards based on empirical research aiming to reduce criminal activities. Within the criminal landscape, organized groups deploy IEDs along roads and in areas surrounding illicit crops to obstruct the manual eradication of coca plants and inflict harm on civilians, military personnel, police officers, and their specialized dogs; therefore, the CNP uses explosive detection dogs (EDDs) trained for operational demining (17) and to counteract terrorist actions by outlaw groups. Furthermore, existing research supports odor discrimination in canines, including mechanisms of chemical signal perception, olfactory memory, and the effects of scent contamination during training and certification for illegal substance detection (18, 19). Other studies in canine forensic detection have explored the use of dog teams to detect human remains and residual odors from concealed bodies to hinder criminal investigations, considering variables such as soil type, temperature, and humidity (20). Research has also encompassed the use of dogs in locating missing people during natural disasters (21) and in detecting corpses, anatomical fragments, and biological fluids at crime scenes (22), as well as new proposals that enhance security and defense through canine units (23). More recent work involving human analog models (*Sus scrofa*) has examined canine performance for decomposition residual odor detection (24), along with identifying the volatile organic compounds released when a body is moved (25).

Research on CDDs is valuable for both the management of threatened species and invasive-species eradication programs, as it enables evaluation of accuracy, sensitivity, effort, and cost, and comparisons with other detection techniques (26). Furthermore, CDDs rely on their exceptional olfactory abilities; they can search more quickly, cover larger and more varied terrain (27), and locate more samples than humans and other analytical tools. These lines of research help refine standards for working dogs operating in complex environments linked to transnational organized crime and profitable criminal activities such as drug trafficking and wildlife trafficking (28). However, outcomes may vary depending on methodological standardization, odor discrimination criteria, and protocols used (1, 29). The effectiveness of detection dogs is not guaranteed; it depends heavily on careful selection of the dog and handler, rigorous training, appropriate sample and scent-handling protocols, and environmental conditions such as terrain and the odor target (30). In addition, anthropogenic changes continue to threaten species and their habitats. In this context, when properly implemented, canine detection represents a powerful, non-invasive, and cost-effective approach for surveying species, monitoring endangered populations, supporting law enforcement against wildlife trafficking, and contributing to biodiversity conservation. It also complements other techniques by overcoming some limitations of camera traps alone (31, 32).

Internal information of CNP based on data regarding illegal wildlife trafficking indicates that species most trafficked in Colombia include the Icotea Turtle (*Trachemys callirostris*), Morrocoy Turtle (*Chelonoides carbonaria*), Green Iguana (*Iguana iguana*), Orange-chinned parakeet (*Brotogeris jugularis*), Common Parrot (*Amazona ochrocephala*), Cheja Parakeet (*Pionus menstruus*), Squirrel (*Notosciurus granatensis*), Gray Marmoset (*Saguinus leucopus*), White-fronted Capuchin (*Cebus albifrons*), and Poison Dart Frogs (*Dendrobatidae* spp.). Between 2017 and 2024, the CNP carried out a total of 62,706 seizures, with canine detection teams participating in 8,166 incidents. Smugglers transport and sell various animals using improvised methods, subjecting them to stressful conditions that frequently lead to death. Despite the efforts of organizations, foundations, and both state and private entities, 80% of seized animals do not survive. Several species are taken to quarantine sites, where they receive first aid and proper habitat management (33). In response to the rise in wildlife-trafficking-related crimes, the CNP launched a pilot study in 2012 to conduct a detailed evaluation of training aids and to develop training and certification standards for this detection discipline. In Bogotá alone, 180 operations conducted in 2024 led to the seizure of 3,634 animals, highlighting the operational effectiveness of specialized canines in combating wildlife trafficking. Thus, the objectives of this study are (1) to develop standardized procedures for wildlife odor training aids and scent banks, and define certification processes within the CNP and (2) to determine dogs’ sensitivity and specificity in discriminating between target and decoy scents through a double-blind test during the certification of animal scent detection dogs.

## 2 Materials and methods

As part of this project, 22 dogs, preselected by the suppliers, participated in this study, comprising 11 females and 11 neutered males aged 12–24 months (mean ± standard deviation [SD]: 18.52 ± 3.21 months). All were evaluated by instructors from the Colombian Police Canine Guide and Training School (19) and weighed 15.5–30.0 kg (mean ± SD: 24.29 ± 2.94 kg). The dogs belonged to the following breeds: Labrador Retriever (*n* = 13), German Shepherd (*n* = 5), and Belgian Malinois (*n* = 4). Of the 22 dogs proposed by the supplier, 6 were selected, each with a score of at least 4/5. During the first week, the six variables were evaluated, refining impulses, understanding each dog’s behavior when faced with different stimuli and performing the proposed exercises. Of the 6 dogs, one had respiratory problems (laryngotracheitis) and was discarded from the study. One dog withdrew from the test due to a loss of focus during sniffing, which affected its perseverance, leaving just 4 dogs for the study (1 male German Shepherd, 1 female Labrador, 1 male Labrador, and 1 male Belgian Malinois).

**Figure 1:**
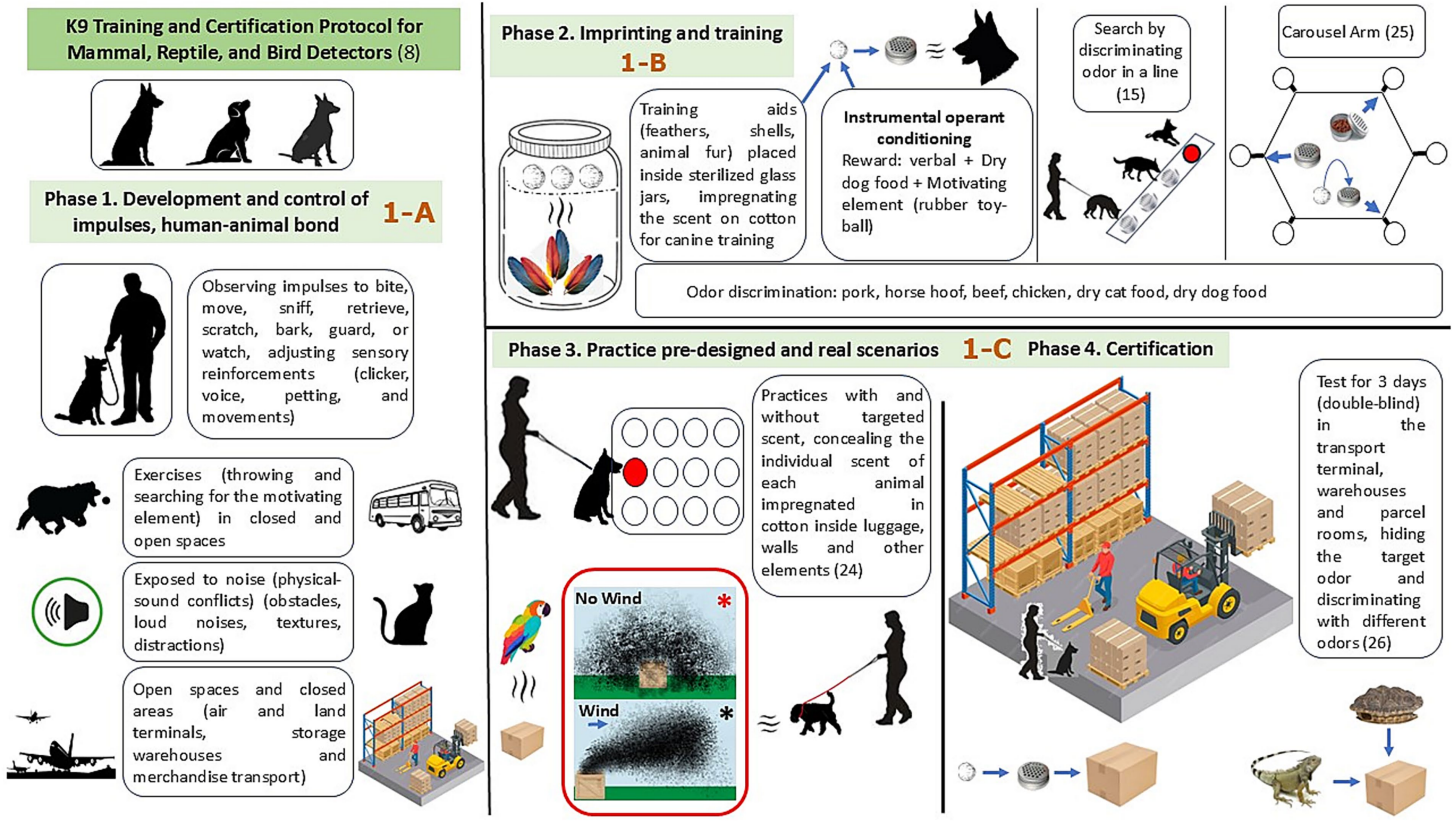
Protocol for selecting the human–animal bond, training, and certification of K9 teams that detect mammals, reptiles, and birds. If there is no wind, scent spreads evenly in all directions, forming a spherical scent pool around the hide (*). Basic scent cone with slow, constant wind (*). Standards and protocols for the management of training aids, and for single-blind and double-blind testing. Additionally, performance types, standard assessments, and operational scenarios (19, 26, 35, 38, 56). 1-A: Adaptation to real-life scenarios. 1-B: Odor preparation and standardization, imprinting and training. 1-C: Practice pre-designed and real scenarios and certification. Red asterisk no wind, black asterisk wind.

The study was conducted over 25 weeks (Figure 1), following established protocols for odor discrimination (7, 18) and carried out in four stages. The target species for wildlife detection included mammals, reptiles, and birds. Assessments were conducted in both open areas and interior spaces, including as airports and bus terminals, as well as warehouses for cargo storage and transportation. These locations were exposed to various distractions, including noise and the presence of other animals (in Colombia, live animals, such as chickens and rabbits, are sold in cages). Additionally, distractor odors were used to introduce scents from species such as pork, horse, raw beef, chicken feathers, cat food, and dry dog food. The canine assessments took place in Bogota, Colombia, at an altitude of 2,650 m above sea level, with an average temperature of 16 °C, and a relative humidity of 70%.

### 2.1 Odor preparation and standardization

Depending on the animal species (reptile, mammal, or bird), specific training aids, free from other odors, must be available and donated by animal shelters. As part of the protocol, sterile, 3-L glass jars with wide screw-top openings must be fitted with a mesh screen at the top. Cotton is placed in the mesh, and subsequently, the turtle shell, bird feathers, and reptile scabs are added (Figure 1B). The training aids must be collected in accordance with strict standards and prepared by laboratory personnel wearing sterile gloves. The glass jar must be sealed and labeled with the collection date and the scientific name of the species. These jars were transported to the laboratory where the training took place and handled only by the trained and qualified technician, who also verified and maintained the storage conditions (specifically the humidity and temperature in the metal shelving where glass jars are stored). The training aids are impregnated for 2 weeks to allow the odor of each target species to impregnate the cotton substrate. Daily, the scent-impregnated cotton is extracted and placed in small metal cans with small openings that allow the target scent to escape (Figure 1B). At the beginning of training, each handler learns about the Instrumental Operant Conditioning: verbal reward, dry dog food, and motivating element (rubber toy/ball). As training progresses, the learning curves of both the handler and the dog improve (the instructors decide whether the dog and its handler need additional training). The impregnated cotton and the small metal cans are replaced after each training session. Therefore, each person on the interdisciplinary team (laboratory technician and sample handling personnel) (handlers, instructors, data collectors, and evaluators) fulfilled specific roles in each part of the process and adhered to the standards of the International Organization for Standardization (ISO), General Requirements for the Competence of Testing and Calibration Laboratories, ISO/IEC 17025:2017 (34), in addition to the criteria for odor discrimination (18) and substance handling (19). Training aids were selected based on the high trafficking rates of reptiles, birds, and mammals in Colombia, as well as the availability of animal protection organizations that could supply these materials. It should be noted that live animals were only present in week 5 and for 3 days during the certification process, handled by veterinarians and expert technicians, and came from previous seizures of domesticated animals to ensure their wellbeing and health during handling.

### 2.2 Phase 1: impulses development and control (weeks 1–4)

Achieving a functional synergy (human–animal bond) between the handler and their canine for 4 weeks, with the aim of perfecting impulses and understanding their behavior when confronted with different stimuli, performing retrieval exercises (throwing and searching for the motivating element) in closed and open spaces, and in the face of physical-sound conflicts (obstacles, loud noises, textures, and distractors). Similarly, the basic needs of each specimen were met by observing the impulses to bite, move, sniff, retrieve, scratch, bark, guard, or watch, adjusting the sensory reinforcements (clicker, voice, caress, and movements); material reinforcement (such as towels, balls, and food) culminates in the formation of a strong bond within the canine team, resulting in two types of relationship: (1) material reinforcement, called physical and (2) affective reinforcement resulting from the hierarchical and emotional interaction between the handler and the animal. During this stage, adaptation to real-life scenarios such as ports, airports, bus terminals, marketplaces, and supply and transportation sites, where illegal trade of mammals, reptiles, and birds commonly occurs, was sought (Figure 1A). Dogs were selected based on a 2 × 2 contingency table score on a scale of 15. The pair that achieved at least 4/5 in the first week maintained the synergy between the handler and their dog for an additional 3 weeks. Of the 6 dogs, one had respiratory problems (laryngotracheitis) and was discarded from the study. One dog withdrew from the test due to a loss of focus while sniffing, which affected its perseverance, leaving just four dogs for the study (one male German Shepherd, one female Labrador, one male Labrador, and one male Belgian Malinois). The following variables were evaluated: (1) Latency to regain motivating stimulus, (2) Focus during odor detection, (3) Human–animal bond, (4) Level of attention and expectancy, (5) Search dynamics, and (6) Search endurance (see Table 1).

**Table 1:** Variables assessed in each canine and Phase 1 score: impulse development and control, m/20 s (distance traveled in meters per 20 s).

S. No.	Parameter evaluated	Exercise test	Score
1	Latency to regain the motivating stimulus	10–20 m/20	1
		20–40 m/20	2
		40–60 m/20	3
		60–80 m/20	4
		80–100 m/20	5
2	Focus during odor detection	The canine does not sniff	1
		The canine sniffs and withdraws	2
		The canine sniffs and remains	3
		The canine sniffs and fixates on the substance	4
		The canine sniffs, fixates, and tries to remove the substance	5
3	Human–animal bond	Does not respond to the handler’s call	1
		The dog watches the handler, but moves away	2
		The dog responds to the handler’s call, but moves away	3
		The dog returns to the handler’s call	4
		The dog returns to the handler’s call and allows competition	5
4	Level of attention and expectancy	Expectation is maintained during training for 2 min	1
		Expectation is maintained during training for 3 min	2
		Expectation is maintained during training for 4 min with motivation	3
		Expectation is maintained during training for 5 min with motivation	4
		Expectation is maintained during training for more than 6 min without motivation	5
5	Search dynamics	Begins searching and loses interest	1
		Begins searching and quickly loses interest	2
		Begins searching enthusiastically, but becomes interested in other scents (e.g., urine marking)	3
		Begins searching enthusiastically and continues for 3 min	4
		Begin searching enthusiastically and continue for 6 min	5
6	Search endurance	The canine searches for 1-min periods and abandons the search	1
		The canine searches for 2-min periods and abandons the search	2
		The canine searches for 5-min periods and observes the handler	3
		The canine searches for more than 5 min until finding the chew toy	4
		The canine searches for more than 15 min	5

### 2.3 Phase 2: target odor impregnation (weeks 5–16)

Once the impulses were balanced and the canine team bond was established, the target odor for each species was imprinted using cotton-impregnated pads. Feathers, hair, and skin from mammals, reptiles, and birds were placed in sterilized glass jars. At the beginning of the discrimination training, the scent was imprinted on cotton, placed in metal cans with holes. When the dog inhaled and exhaled, he was immediately given a verbal reward, added to a reward of dry dog food, and delivery of motivating element (rubber toy/ball) (see Table 2). In this second phase, the odor-impregnation and discrimination variables were evaluated during training, based on the number of repetitions in each daily and weekly session. This variable was used to establish the canine’s attention focus and interest in the target odor. The canine was allowed to express the strongest signal (sitting or lying down) when reaching the target odor and was reinforced in each session. Additionally, training exercises were performed on a six-arm scent discrimination wheel (35) and using a wall system (26), which facilitated the learning of target odors at different heights through a systematic process. Training began with the introduction of the first target odor—parrot feathers—into the canines’ olfactory memory. Trainers presented the odor, reinforced correct responses verbally, and rewarded successful identifications with a chew toy. Each dog performed one exercise at a time, with the exercise repeated across an average of five trials. To build robustness, training scenarios were systematically varied, enhancing search behavior, alertness, and fixation on the target odor. Task complexity was gradually increased by incorporating a six-arm wheel and a six-hole wall, followed by sequential exposure to additional species odors—feathers from the *Ognorhynchus icterotis* and *Ara macao*; hairs from the *Saimiri sciurus*; carapace of the *T. callirostris*; skin from the *Boa constrictor*; and scales from *Crocodylus fuscus* and *I. iguana*—trained as target odors through positive reinforcement, as was used for training parrot feathers. Advanced exercises employed two scent wheels at different heights and a 12-hole wall, as well as chained searches involving multiple odors or species. Non-target odors, including chicken feathers and cat food, were subsequently introduced to train the dogs to discriminate target odors from irrelevant stimuli, thereby improving detection accuracy under realistic operational conditions.

**Table 2:** Distribution of training weeks and repetitions for wildlife-detection canines.

Week	Exercise performed
Week 5	Adaptation and approach to scent sources and live animals: shell, skin, fur, and feathers, corresponding to species such as turtles, boa constrictors, monkeys, baboons, iguanas, and birds (parrots and macaws). Ten repetitions in each session (the dog smelled a metal can using a verbal and dry food reward only).
Week 6	Four repetitions of scent sniffing per session, with three sessions daily. Search by discriminating odor in a line.
Week 7	Search by discriminating odor in a line. Five repetitions of the scent strengthen the fixation of sitting or lying down behavior*.
Week 8	Induction to search vehicles, suitcases, and rooms, with four repetitions; one for each scent, whether feathers, snake scales, monkey fur, or turtle shell.
Weeks 9–12	Using a spinner and a wall, the behavior is established. Nine repetitions are performed, divided into three sessions of interspersed work (one canine performs three repetitions; then the second canine enters, continuing this sequence).
Weeks 13–16	Induction to search vehicles, suitcases, and rooms, with four repetitions, one for each odor (feathers from the *O. icterotis* and *A. macao*; hairs from the *S. sciurus*; carapace of the *T. callirostris*; skin from the *B. constrictor*; and scales from *C. fuscus* and *I. iguana*; the lying-down behavior* in response to the odor is strengthened).

*The down or sit behavior depended on the strongest signal emitted by each dog and was reinforced for each exercise individually. Three sessions per day per dog, depending on the number of repetitions per week.

To enhance odor recognition and generalization across contexts, training was conducted in varied environments, such as open fields or enclosed spaces. It included challenges such as cotton-impregnated training aids with the odor of non-target animals (e.g., horse or pig hooves) and different food odors (e.g., raw beef, chicken feathers, cat food, canned food, or dry dog food). The scent discrimination task was performed on a 6-arm multiple-scent wheel to assess the dogs’ training, using both blanks and the target odor. Training sessions were conducted to analyze the behavior and effectiveness of olfactory memory conditioning. For dogs showing false-positive or false-negative responses, corrective training based on direct odor–reward association was applied. This involved placing the odor near the dog’s nose and immediately rewarding it with food. A change in behavior was noted when the dog was praised with the phrase “good dog” repeated 10 times. Cotton balls impregnated with the odor of each species served as the target odor, which was replaced after each training session (Figure 1B).

### 2.4 Phase 3: practice in pre-designed and real-life scenarios (weeks 17–24)

This phase was divided into an initial stage of controlled searches in pre-designed scenarios and a recording stage in a real-life scenario to detect the animal species to be identified for wildlife trafficking control (see Table 3). Furthermore, introductory field practices were intentional and gradual, concealing each animal’s individual odor impregnated on cotton balls inside luggage and other items. The dog was encouraged to search for this odor directly and was rewarded when it responded “sit” in front of the item containing the odor-impregnated training aids (cotton balls). Furthermore, the dog was exposed to several objects that did not necessarily contain the odor, concealing live wildlife species in luggage, packages, and cargo holds without causing stress to the target animal. Similarly, the odor of other domestic animals was concealed to serve as a distractor, helping to enhance the dog’s olfactory abilities, focus, and concentration on locating the target species (Figure 1C).

**Table 3:** Activities and repetitions for odor discrimination in practice for CDDs.

Week	Activity and repetitions to discriminate against the smell of animals
17–20	A conflicting odor discrimination activity was conducted, with three repetitions. Practices were conducted at the ground transportation terminal, including the vehicle, baggage, and cargo areas.
21–24	Cotton impregnated with the odor of parrot and macaw feathers, snake scales, turtle shells, monkey hairs, iguana skin, and baboon skin. Three repetitions were also conducted daily. Practices with and without targeted scent, concealing the individual scent of each animal impregnated in cotton inside luggage, walls, and other elements.

### 2.5 Phase 4: certification (week 25)

A double-blind test was conducted in the transport terminal, warehouses, and parcel rooms, masking the target odor and discriminating against different odors, according to the parameters of Phase 3 (Figure 1C). A double-blind test is one in which neither the handler nor the evaluator knows the locations of the samples within the operational search scenarios. During the double-blind evaluations, all target scents were intentionally placed by the researchers, and no unplanned alerts to unknown or unplanted materials were recorded. When dogs indicated a target, the presence or absence of the planted sample was verified by evaluators through direct inspection, ensuring accurate classification of true-positives and false-positives. No additional independent screening methods (e.g., chemical analysis or third-party inspection) were used during testing. Therefore, true negatives were defined as samples with the controlled absence of target scents. While this controlled design strengthens internal validity, it may not fully replicate operational environments where unknown or residual odors could be present. This limitation should be considered when interpreting sensitivity and specificity, as real-world conditions may introduce additional sources of false-positives or undetected true targets. Furthermore, the evaluation consisted of three daily tests over three consecutive days. Candidates were determined as fit or unfit for police service based on each canine’s responses, determining the level of agreement between evaluators (Cohen’s Kappa): Poor (0.00); Mild (0.01–0.20); Acceptable (0.21–0.40); Moderate (0.41–0.60); Considerable (0.61–0.80); and Almost Perfect (0.81–1.00), and only dogs achieving agreement in the “Considerable” to “almost perfect” range (Kappa > 0.61) were considered for certification (36). It should be noted that live animals were present for only 3 days during the certification process, handled by veterinarians and expert technicians, and came from previous seizures of domesticated animals to ensure their wellbeing and health during handling.

### 2.6 Statistical design

The Statistical Package for the Social Sciences (SPSS) software was used. For the phase 1 test: impulse development and control, a univariate analysis was performed using 2 × 2 contingency tables and the Fisher test. For phases 3 and 4 tests, the dog’s olfactory performance was determined: (1) True positive: the dog indicates the target odor in the manner in which it was trained (response “sit or lie down”), (2) False-positive: the dog alerts in a non-target position (control), (3) False-negative: the dog does not exhibit the trained alert in the presence of the target odor, and (4) True negative: the dog does not alert in the absence of the target odor, establishing the usefulness of the tests for each canine^1^ (37). The dog’s sensitivity (true positive rate; Equation 1) and specificity (true negative rate; Equation 2) were calculated using the observed counts of true positives, true negatives, false-positives, and false-negatives (38).

Equation for sensitivity:


$$\text{Sensitivity}=\frac{\#\text{True Positives}}{\left(\#\text{True Positives}+\#\text{False Negatives}\right)}$$ (1)

Equation for specificity:


$$\text{Specificity}=\frac{\#\text{True Negative}}{\left(\#\text{True Negative}+\#\text{False Positive}\right)}$$ (2)

In addition, the level of agreement among the evaluators of the double-blind test for phase 4 certification was assessed using Cohen’s Kappa, and the association between the partial results of phases 3 and 4 and the certification result (Pass or Fail) was determined. The analysis aimed to evaluate test performance by plotting sensitivity against specificity, identifying false-positives and false-negatives, and determining the discriminatory power of each test.

## 3 Results

### 3.1 Phase 1: development and control of impulses (weeks 1–4)

Of the 22 dogs proposed by the supplier, 4 passed the tests outlined in Table 1 and were selected with scores of at least 4/5. During the first week, the six variables were evaluated, refining impulses, understanding each dog’s behavior when faced with different stimuli and performing the proposed exercises. As a result, only 4 dogs (18%) completed the first phase with positive outcomes, working effectively with their handler over the 4-week evaluation period. Additionally, four factors led to the exclusion of other dogs: attention and expectation/time issues ruled out 6 dogs; poor search dynamics (≤3 points) eliminated 10 dogs; one dog was ruled out due to behavioral problems (loss of focus), and another dog was ruled out due to respiratory illness. It was noted that although these dogs started the search with enthusiasm, they became distracted by other scents (e.g., urine marking) or lost interest (withdrawing within the first 4 min), which diminished their focus on the target scent.

### 3.2 Phase 2: target odor impregnation (weeks 5–16)

Imprinting refers to odor discrimination training, in which the dog is trained to be rewarded for sniffing or finding a particular odor (38). Following the protocol outlined in Table 2, the 4 dogs performed 719 repetitions each over 12 weeks (weeks 5–16) to imprint the odor. If the dogs had more than three doubts each day, they were reinforced with 10 additional repetitions (the dog smelled a metal can using only a verbal and dry dog food reward). with subsequent discrimination on the wall with other odors as a control, and verbal reward, plus food and a toy on only two occasions. This was necessary because 2 of the dogs failed to recognize the new odor of *B. constrictor* (snake scaling) during week 10 and doubted when they were prompted to search vehicles, suitcases, and rooms. Subsequently, in week 14, the 4 canines were evaluated again, discriminating against and responding positively to the new odor, with reinforcement continued until week 16. It is evident that there was a significant association between alertness (sitting or lying down) and the presence of the target odor during the criterion tests (Fisher’s exact test, *p* < 0.0001). The six-arm scent wheel odor discrimination test showed that the dogs did not exhibit false-positive or false-negative signals, with negative predictive values (NPV) of 0.11 or positive predictive values (PPV) of 0.94, establishing the usefulness of the test for each canine.

All four dogs showed rapid improvement over the first 3 weeks, increasing with each week until week 16. Sensitivity and specificity were calculated for each day (15 repetitions per day). When a dog had doubts about other odors (horse odor, dry dog food, cat food, pork odor, beef, chicken feathers), they were noted as incorrect (false-positives). All four dogs had at least one session where both measures were at or above 90%.

**Figure 2:**
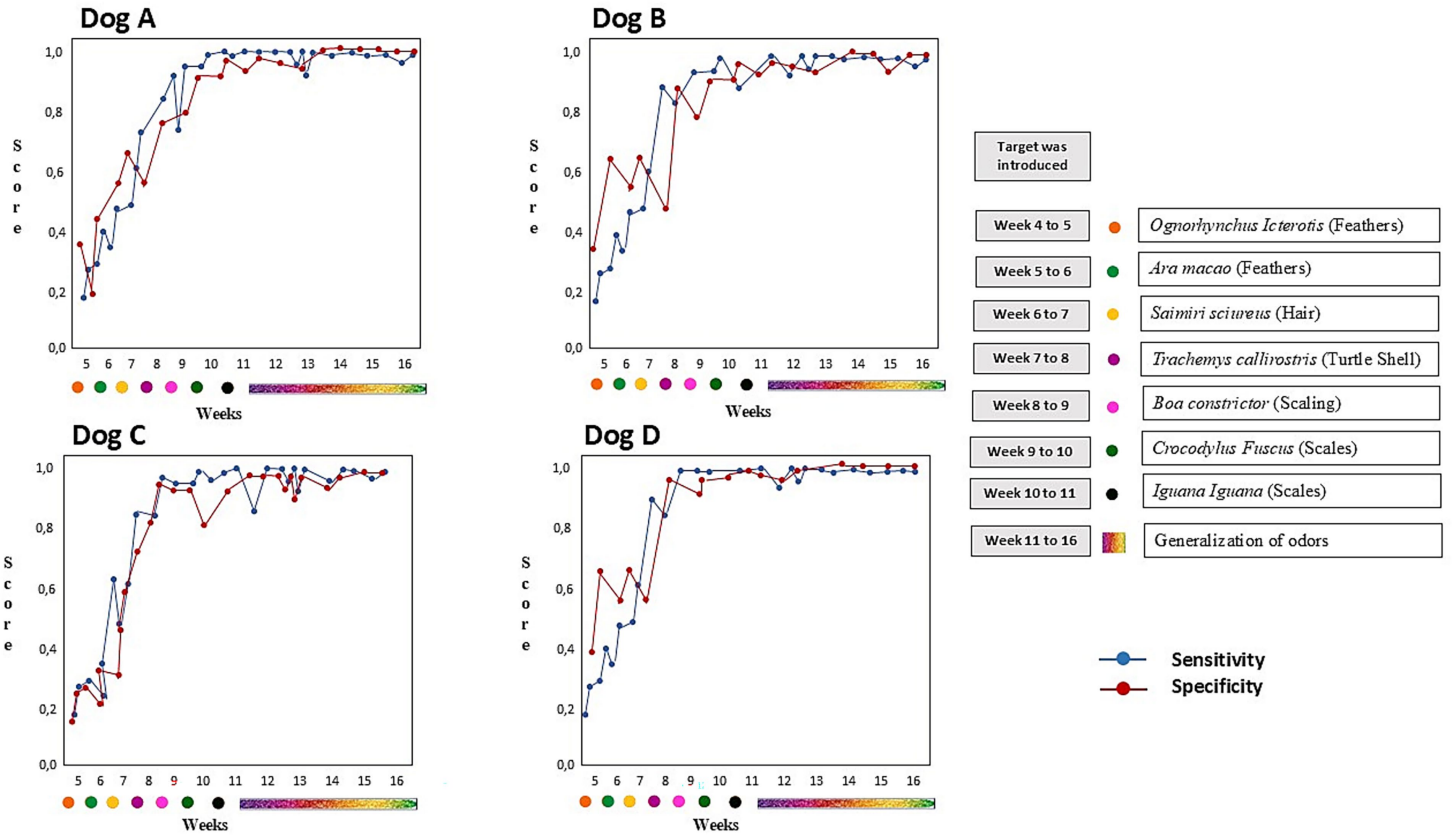
Sensitivity and specificity of four dogs during odor discrimination and target introduction over time.

Averaging all training sessions together, the dogs showed a high level of sensitivity (Dog A—92.4%, Dog B—95.7%, Dog C—94.6%, and Dog D—93.2%) and specificity (Dog A—93.6%, Dog B—94.7%, Dog C—95.6%, and Dog D—96.7%) (Figure 2). However, there were no differences between the four dogs in their levels of consistency for the scents of *O. icterotis* (feathers), *A. macao* (feathers), *Saimiri sciureus* (hair), *T. callirostris* (Turtle shell), *B. constrictor* (Snake scaling), *C. fuscus* (scales), *I. iguana* (scales) across training. Dogs A and B had 5 total false-negatives compared to 9 and 10 for C and D, respectively.

### 3.3 Phase 3: practice in pre-designed and real-life scenarios (weeks 17–24)

**Figure 3:**
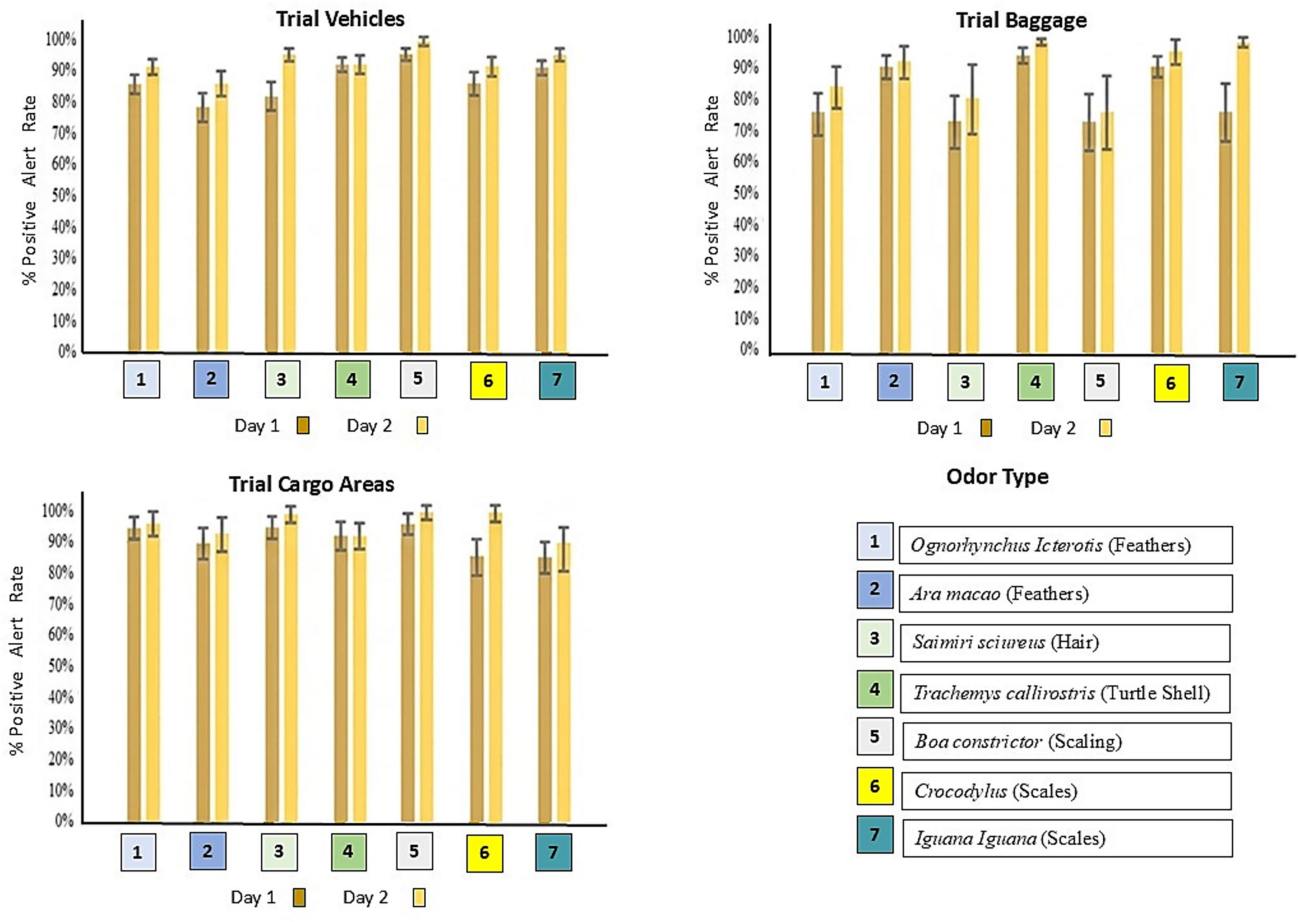
Comparison of performance between the first and second day of the trial (week 22), for the average across the 4 dogs for each of the three search environments, categorized by odor type: feathers from the *O. icterotis* and *A. macao*; hairs from the *S. sciurus*; carapace of the *T. callirostris*; skin from the *B. constrictor*; and scales from *C. fuscus* and *I. iguana*. Average % positive alert rate ± standard error (SE), confidence interval (95%).

In the first 2 days of week 17, all four dogs showed false-positives, particularly with the odor of a pig’s hoof. These were corrected, reinforced with 10 additional repetitions when the dog smelled metal using a verbal and a dry food reward, followed by discrimination on the wall with other odors as a control. The dogs then met the criteria (Table 3). Additionally, they successfully discriminated between odors during practice and controlled searches in pre-designed scenarios (vehicles, baggage, and cargo areas). Generally, a higher positive alert rate was observed on the second day of the trial, with accuracy rates ranging from 5 to 99% (Figure 3). Similarly, the performance was compared between the first and second day of the trial (week 22), categorized by odor type: feathers from the *O. icterotis* and *A. macao*; hairs from the *S. sciurus*; carapace of the *T. callirostris*; skin from the *B. constrictor*; and scales from *C. fuscus* and *I. iguana*, evaluating the % positive alert rate.

### 3.4 Phase 4: certification (week 25)

**Figure 4:**
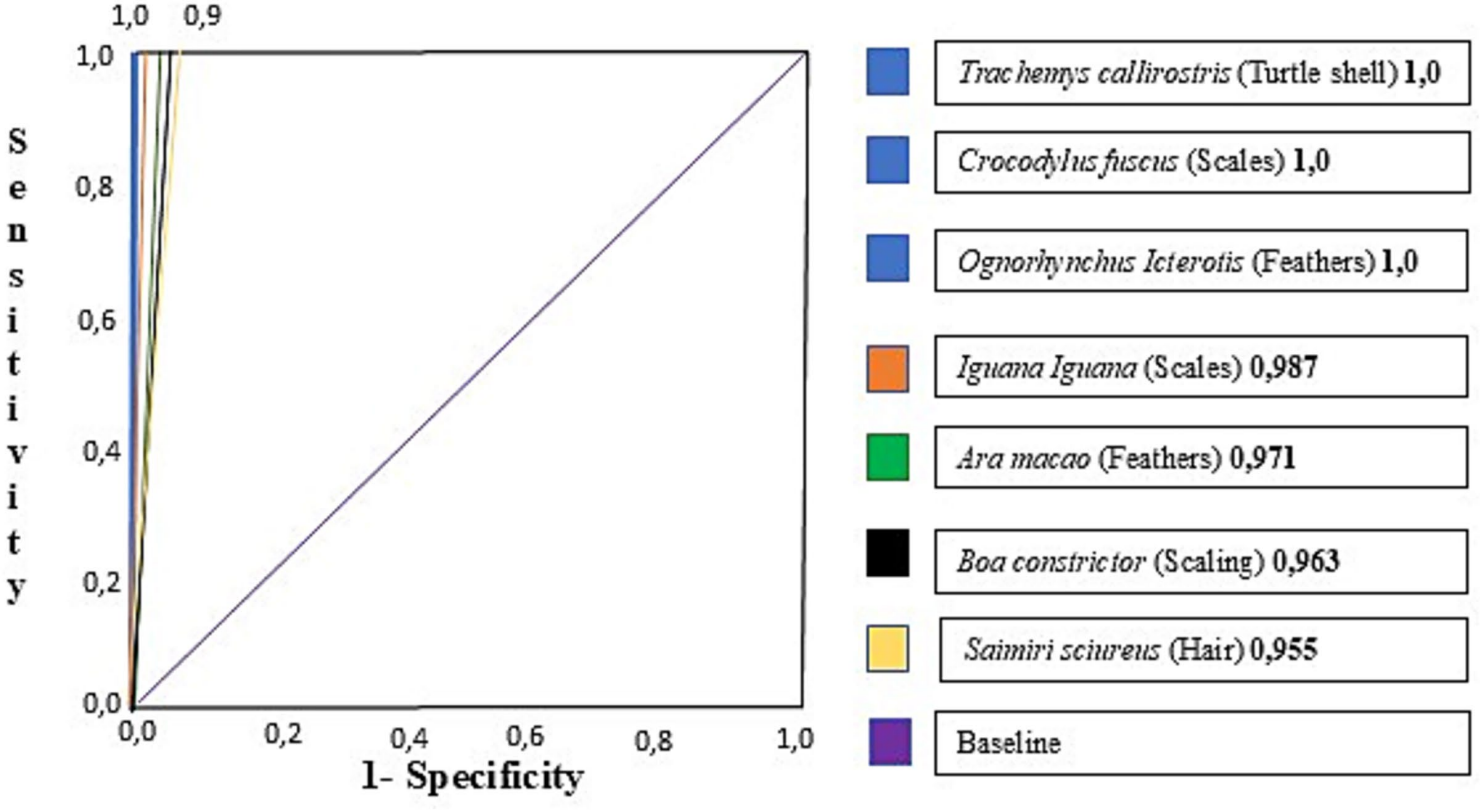
Receiving operating characteristic (ROC) curves to estimate the specificity of the certification test during 3 days.

The double-blind test conducted at the ground transportation terminal, vehicle parking, baggage claim area, and warehouses determined that handlers and evaluators had a near-perfect agreement (Cohen’s Kappa) (0.95–1.00) for all odors. A significant association was observed between alertness and the target odor (Fisher test *p* < 0.01). The four canines were certified by presenting true positives during the three daily tests in the three consecutive days and discriminating the trained odors (feathers from the *O. icterotis* and *A. macao*; hairs from the *S. sciurus*; carapace of the *T. callirostris*; skin from the *B. constrictor*; and scales from *C. fuscus* and *I. iguana*) (Figure 4). Similarly, no false-positives or negatives were obtained, identifying the behavioral changes in the canines. There was no warning signal or behavioral change rewarded by the handler that indicated a false-positive or negative for the distracting odors of horse odor, dry dog food, cat food, pork odor, beef, or chicken feathers. A third (non-blind) observer was present to indicate whether a reward or reinforcement should be administered upon identification of an indicative behavior.

## 4 Discussion

There are significant markets for the trafficking of endangered species and various transit sites for the illegal wildlife trade worldwide, which require stricter border and policy control procedures (39). The cross-border bio-crime cooperation model introduces a novel approach to replicating the cooperation framework established by the trial of veterinary public health, justice, and law enforcement/customs, utilizing international police and customs cooperation centers as a connecting link between public entities in neighboring countries (40). Therefore, the results of the number of seizures and captures help characterize the criminal groups involved in the crime, their operations, the exploitation of citizens, and strategies such as the use of animal-detection dogs to detect these species in different contexts. This operational need underscores the importance of standardized, evidence-based training protocols to ensure reliable canine performance in real-world enforcement scenarios. The Phase 1 four-week work allowed the dogs to refine their impulses and understand their behavior when confronted with different olfactory and auditory stimuli. The focus on sniffing was the variable that enabled the dogs’ perseverance to be identified in each of the proposed exercises. These results are consistent with a study of 423 dogs undergoing an exhaustive selection process in Colombia in which the perseverance variable was found to be fundamental. Despite this, 2.8% (12/423 dogs) failed the final certification test (19). Furthermore, scent discrimination through double-blind testing using reward mechanisms (scent wheel) determines changes in specific behaviors in each dog (alertness), and the focus on the trained odor allows for a human–animal bond that contributes to the canine team’s experience. In addition, the certification of this type of pairing is carried out in real-life settings where illegal animal trafficking is prevalent. Detection dogs are frequently evaluated in “line-up” or “scent recognition” procedures (items such as suitcases, paint cans, etc., are placed in a line or circle, and the dog is asked to search them) because they are inexpensive, easy to set up, and adaptable to different environments (41, 42). Although double-blind testing was implemented in this study and is recognized as a robust and rigorous approach, it is inherently time-consuming and labor-intensive. In real-life operational scenarios such as warehouses, market squares, or parcel sites, additional procedures are required to manage potential extraneous cues that may influence canine behavior, including residual scent markings from previous samples, memorization of odor order across repeated trials, and unintentional auditory cues. These challenges necessitate careful coordination among evaluators to conceal target odors (feathers, fur, hair, and other materials) and the use of strict randomization protocols to ensure reliable odor discrimination. Importantly, while fully automated systems may further standardize testing conditions, the present methodology maintained scientific rigor under operational constraints and did not compromise the validity of the data collected (43).

A statistically significant association between alertness and target odor further supports the idea that the canines’ behavior was directly influenced by the target odors. This suggests that not only do the dogs exhibit strong scent discrimination (44), but their cognitive engagement and alertness are enhanced when they encounter the target odors, demonstrating their responsiveness to specific cues and their ability to focus on relevant stimuli in complex environments.

The results of the final double-blind certification assessment demonstrate a high level of agreement among handlers and evaluators on the target material being detected by the canine. We found near-perfect to perfect agreement for parrot feathers and monkey hair target material, while considerable agreement was obtained for turtle shell, snake scales, iguana, and crocodile. These results show promising canine detection accuracy and reliability but suggest room for improvement in the detection of specific targets, the strength of the canine indication response, and the handler’s evaluation of these behaviors. These findings align with previous studies showing that the trained canines can exhibit a high proficiency in discriminating between scent differences in two species of beaver samples and in discriminating olfactory signals of beaver species from scent marks (45). For the other target odors—turtle shell, snake scaling, iguana, and *Crocodylus*—Cohen’s Kappa values range from 0.75 to 0.80, indicating a considerable agreement and suggesting that the canines can reliably identify these odors, although with slightly less certainty than the top-tier targets.

One of the most significant findings of this study is the absence of false-positives or false-negatives during certification, underscoring the effectiveness of the detailed canine training regimen. Canines demonstrated an impeccable ability to identify the target odors while avoiding distractions from irrelevant scents, as demonstrated by their consistent behavior across tests. The absence of false-responses indicates that dogs reliably identified the target odors without confusing them with similar odors in the environment. This is crucial in applications such as wildlife conservation, where detection precision is paramount in complex terrain. Overall, while the observed zero false-positive and false-negative rates reflect strong performance under controlled certification conditions, they should not be interpreted as absolute indicators of field performance. Instead, they support the need for continued testing with novel specimens from different individuals and contexts to further evaluate generalization capacity and ensure robust deployment in operational wildlife-detection scenarios.

False-positives for the pig-hoof scent as a decoy occurred on the first evaluation day for two of the dogs, indicating that the introduction of new practices in pre-designed and real-life scenarios may affect the handler’s concentration and understanding of the dog’s particular signals. However, discriminating new scents is a normal part of training, false-positives or false-negatives can occur. At the end of an exercise where the dog detects false-positives or negatives, a reinforcing scent is applied, added to the discriminating odor in a line, and the Carousel Arm to remember the smell and maintain trust on the part of the handler (reinforced with 10 additional repetitions when the dog smelled a metal can using a verbal and a dry food reward) (Figure 1B). Similarly, handlers noted that two dogs were distracted at the start of the trials in week 17, and therefore modified the leash handling, indicating to the dog where to sniff and search to keep the dog focused and registering the boxes, consistent with another study, where false indications were also noted on empty controls, after the dog had searched every container (46). False indications following a blank search may be associated with the detection dog failing to find a target and taking the chance of getting a reward by displaying a false-positive indication on a blank pipe (47). Due to the above, the handler became more confident in reading the detection dog’s behavior as the trials progressed during the trial. False indications were unrelated to the position of the training aids impregnated with the scent of each species, rendering residual-scent indications unlikely given the impeccable handling of the training aids and their placement in strategic locations within vehicle, baggage, and cargo areas, thereby avoiding secondary contamination with other odors.

The canine certification process, which involved three consecutive days of daily tests, further validated the dogs’ proficiency. By accurately identifying both target odors (parrot feathers, macaw, snake scaling, etc.) and differentiating them from distractor odors (horse, dry dog food, and meat), the canines demonstrated robust training and consistency over time. This consistent performance not only confirms the dogs’ competence in odor detection but also suggests that the training program was effective in establishing reliable scent recognition over the short-term testing period.

The results of this study highlight the high reliability of canine odor detection, with canines demonstrating excellent performance in detecting and discriminating between specific target odors that remained static within a dynamic operational context. The absence of false-positives and false-negatives, and the significant association between alertness and the target odor, strongly support the effectiveness of the training program. However, future work could explore the challenges of detecting moving targets and examine whether adjustments to the training protocol can improve canine performance in more dynamic settings. Although this study did not evaluate core body temperature or environmental conditions (average temperatures between 10 °C and 18 °Cs), wildlife-detection dogs in Colombia work in complex environments with high temperatures that affect their thermoregulation. These sites are home-bred animals with good operational performance, which is why it is important to continue studies evaluating hyperthermia (the main cause of death) and estimating core temperature changes, metabolic activity, and environmental conditions in military working dogs in hot environments (48). Therefore, future studies should evaluate how thermoregulation affects wellbeing to measure their performance. In a study of cheetah (*Acinonyx jubatus*) scavenging dogs in a hot, arid Kenyan habitat, the animals were monitored in extreme temperatures and difficult working environments. It was found that after a full day of work, they exhibited softer feces (higher stress levels and elevated body temperature during routine checks the following morning) (49). These results indicate the complexity of standardizing canine searches, where cooling mechanisms (panting) and evaporation are important physiological parameters that must be measured (50) to avoid heat exhaustion, preserve the dog’s ability to perceive chemical signals, and maintain the canine team’s performance.

The results of this study highlight the high reliability of CDDs, particularly at high altitude under specific humidity and temperature conditions. While the study did not assess the dogs’ core body temperature or the environmental conditions (average temperatures between 10 and 18 degrees Celsius), wildlife-detection dogs in Colombia work in complex environments with high temperatures that affect their thermoregulation, warranting further research. Canine handlers should adapt search and training strategies to their dog’s individual paces and limitations, and take environmental influences into account. For example, to find terrestrial amphibian species facing ongoing habitat loss and requiring effective conservation measures, these strategies are useful in areas with dense vegetation and for covering larger regions more efficiently, with success rates depending on favorable climatic and habitat conditions, such as warmer temperatures and sufficient humidity (51). Increased temperatures can increase fatigue and unresponsiveness in dogs, affecting their sense of smell. Furthermore, elevated temperatures can reduce humidity and increase panting (52). Therefore, further investigation of the effects of temperature on canine behavior is needed, especially in pre-designed scenarios. This aligns with the results of our study, where each detection dog exhibited a unique learning curve, and its performance was primarily influenced by its handler’s understanding of the dog’s behavior (human–animal bond), wind (the search is more fluid in enclosed spaces due to the direction of air currents), and favorable environmental factors that allowed the dog to more efficiently regulate its body temperature, improving the learning process, detection performance, and odor generalization. Further studies are needed, especially in countries with varying ambient temperatures, such as Colombia.

Other parameters that can affect searches during physical and cognitive tasks include sudden changes in sound and light (e.g., flashing police sirens and beacons), which are common components of the working environment for working dogs (53). Contrary to the Colombian study, none of the four dogs were affected by loud noises (physical–sound conflicts), possibly because the prior selection process discarded dogs with weak temperaments (19). While dogs selected for their high perseverance and search motivation are more likely to be rated high in overall detection performance (54), the results demonstrate that 72% of dogs are rejected for low perseverance.

Overall, the findings of this study provide evidence that sniffing behavior can be used to effectively assess olfactory alerting performance in dogs, discriminating against the scent of various animal species. Therefore, studies that standardize canine training using real target materials (animal specimens) contribute to better differentiation and generalization of target odors, thereby optimizing performance in real operational scenarios and supporting the evaluation of detection reliability. Such approaches also facilitate the development of structured training tools and associated odor profiles (55), including training aids such as skin, feathers, hair, and shells used in this study. However, these materials are not readily available due to limited supply and regulatory restrictions on trafficked species, which necessitate the use of certified training odors (odor banks) established through strategic partnerships between wildlife reception centers and organizations responsible for canine training programs. This would reduce costs and ensure a consistent supply of trained canine teams. Although the sample size was only four dogs, other studies using CDD have similarly limited numbers of animals (46, 51). The preliminary findings of our study demonstrate a rigorous selection process (only 4 of 22 dogs began phase 1), and effective coordination and teamwork between the handler and the dog over 4 weeks. This involved refining the dogs’ instincts and understanding their behavior in response to various external stimuli in real-world scenarios where illegal trade in mammals, reptiles, and birds commonly occurs. These results are similar to those obtained in other studies that reduced sample size due to the use of live animals for training (51). Future controlled studies should explore this aspect in greater depth due to limitations in live animal training, the protected status of the animals, and the use of training aids derived from protected or critically endangered species, which limits the number of dogs and generates high associated costs. However, during week 5, live animals were used for adaptation and approach to odor sources, and during the last 3 days of the certification test, domesticated animals (animals that previously lived with humans for many years) were used, making it impossible to reintroduce them to their habitat. The specimens never had contact with the dog or handlers, and ethical, health, handling, and animal welfare standards were guaranteed.

External factors such as temperature, air currents, access to the site, and humidity directly affect the search for the target odor in real-life scenarios in human remains detection dogs (20), hence the importance of continuing to evaluate the dog’s behavior and response to external factors that generate stress for the dog and its handler during the search. Similarly, other aspects of dog behavior with respect to olfactory detection and alert response should be investigated to identify and standardize parameters, independent of the target odor or the situation presented. Finally, there is a study in the empirical literature on best practices for the evaluation, selection and improvement of working dogs (56, 57), including opportunities for interdisciplinary research to optimize canine welfare (58), which invites to continue with research that evaluates chemical signals, olfactory memory and the influence of olfactory contamination; highlighting the complexity of smell as a sophisticated communication system that must be estimated in the practical field, evaluating the performance of canine teams.

## 5 Conclusion

This study highlights the effectiveness and efficiency of using dogs to detect wild animals. During training, it is crucial to focus on target odor discrimination and to reinforce it through systematic repetition to enhance performance and minimize false-positives or false-negatives. When errors occur, it is important to revisit the stimulus–response and reward processes during training. This research is the first to certify individual detection dogs for a variety of mammal, reptile, and bird species, and it should be revisited alongside other studies that assess operational performance. It is recommended that dogs be trained with odors provided by wildlife reception and rehabilitation centers annually. This would enhance standards, support the identification of illegal wildlife trade, and ensure that dogs are re-certified each year, ultimately contributing to both biodiversity conservation and enforcement effectiveness. Additionally, understanding each dog’s individual characteristics and how they relate to behavior will help optimize the selection, training, and management of detection dogs. This could reduce financial costs, streamline procurement processes, and improve detection capabilities in operational environments.

Standardizing the selection, training, and certification of operational results is the starting point for evaluating the efficacy and effectiveness of wildlife-detection canine teams. The responsibility to certify these types of animals and measure their performance contributes to adjusting standards based on crime dynamics, especially when discriminating odors in complex scenarios, across different environmental conditions, and at different locations. This helps validate tests in real-life scenarios where various odors converge (e.g., market squares, ports, land transportation), contributing to odor discrimination. Similarly, depending on the animal species (reptile, mammal, or bird) targeted for scent detection, specific training aids should be available that are free of contamination from other odors, allowing for expeditious training and reducing false-positives or false-negatives. Therefore, the canine should be trained according to the type of target required (e.g., feathers, excrement, fur) or a combination of the substances used during training to achieve strong scent discrimination performance. Furthermore, field tests (controlled records) under balanced conditions are essential so that the handler understands the warning signs, enabling synergy between pairs. There are no specific frameworks for training wildlife-detection canines, but proper management and establishment of training and certification protocols are required, tailored to the objectives of each context. Therefore, it is recommended that 3-liter glass jars with a wide, screw-type mouth are ideal for impregnating cotton or gauze with the target odor. It is recommended not to contaminate animal samples and to maximize data recording during “blind tests,” including odor discrimination from other types of distractor odors (e.g., latex gloves, food, among others), to achieve short-, medium-, and long-term results, to enhance the responsiveness of canine teams, minimize frustration within the team, and achieve progressively higher success rates as experience increases.

Finally, effective public policies in strategic areas, aligned with other relevant issues, should be implemented to avoid the misallocation of resources. These policies can foster an environmental culture that discourages the consumption and trade of wild animals. Within this framework, trained detection dogs represent a key operational tool to support enforcement efforts by improving the identification of illegally trafficked wildlife in transport hubs, markets, and other concealment settings. By engaging national, regional, and local governments, as well as NGOs and justice actors, through educational centers, we can promote alternative regional projects and eco-tourism. Coordinating efforts will help disrupt illicit wildlife trafficking and mitigate the influence of transnational criminal organizations.

## Data Availability

The raw data supporting the conclusions of this article will be made available by the authors, without undue reservation.

## Footnotes

1
https://www.nist.gov/osac/subcommittees/dogs-sensors
